# Which Child Will Benefit From a Behavioral Intervention for ADHD? A Pilot Study to Predict Intervention Efficacy From Individual Reward Sensitivity

**DOI:** 10.1177/1087054720928136

**Published:** 2020-06-11

**Authors:** Myrte J. M. van Langen, Branko M. van Hulst, Miriam Douma, Maarten Steffers, Nicolle M. H. van de Wiel, Els van den Ban, Sarah Durston, Patrick de Zeeuw

**Affiliations:** 1 University Medical Center Utrecht, The Netherlands; 2 Altrecht, Utrecht, The Netherlands; 3 Pro Persona Mental Health, Ede, The Netherlands

**Keywords:** attention-deficit/hyperactivity disorder, reward sensitivity, reward processing, behavioral interventions

## Abstract

**Objective:** This article aims to assess whether individual differences in reward sensitivity can be used to predict which children with attention-deficit/hyperactivity disorder (ADHD) will benefit most from behavioral interventions that include reinforcement. **Methods:** A 12-week behavioral intervention was offered to 21 children with ADHD and their parents. Reward sensitivity was assessed prior to the intervention using a combination of psychological and physiological measures. ADHD symptoms were assessed pre- and posttreatment using the Strengths and Weaknesses of ADHD and Normal behavior (SWAN) rating scale. **Results:** Lower scores on one of the questionnaire scales were associated with greater pre/posttreatment differences in ADHD symptoms. **Conclusion:** We found that pre/posttreatment change was associated with one measure of parent-rated reward sensitivity. Children with low impulsive negative behavior toward gaining reward improved most during treatment. This result suggests that aspects of reward-related behaviors in ADHD may be useful to predict the effectiveness of treatment.

## Background

Behavioral interventions are recommended for the treatment of children with attention-deficit/hyperactivity disorder (ADHD; American Academy of Pediatrics, 2011). These interventions rely on teaching behavioral strategies to parents, teachers or therapists, and on using reward contingencies to reinforce desirable behaviors (Barkley, 2002). Despite their popularity, the effects of these interventions on core symptoms of ADHD have been reported to be moderate at best (Daley et al., 2014; Sonuga-Barke et al., 2013). Interestingly, reward processing is a neuropsychological domain that seems to be affected differentially in children with ADHD (Durston et al., 2011; Luman et al., 2005, 2010). This heterogeneity may therefore provide a clue to the limited effectiveness of behavioral interventions.

Sensitivity to reward and reinforcement has been reported to be changed in children with ADHD compared with typically developing children. Both in behavioral data and in neuropsychological and imaging data, differences have been found at the group level (Durston et al., 2011; Luman et al., 2005, 2010). Children with ADHD respond positively to reward and tend to show greater improvement in task performance following reward than typically developing children (Luman et al., 2005, 2010). Hence, once a reward has been “delivered,” it generates a relatively large response. The anticipation of reward, however, seems to be diminished, which may result in a lower behavioral control of reinforcers in ADHD. Children with ADHD exhibit delay aversion, where they prefer smaller immediate rewards over larger delayed ones compared with typically developing children. (Sonuga-Barke et al., 2008). Similarly, functional magnetic resonance imaging (fMRI) studies have found decreased activity of the ventral frontostriatal reward circuitry, specifically during the anticipation of reward (Durston et al., 2011; Paloyelis et al., 2012; Plichta et al., 2009; Scheres et al., 2007; van Hulst et al., 2017). Neurobiological theories suggest that the dopaminergic system is likely involved; either through a general reduction of synaptic dopamine resulting in reduced sensitivity to delayed reward (Sagvolden et al., 2005; Volkow et al., 2009, 2011), or through what has been termed the dopamine transfer deficit, a reduced firing of dopamine cells in the ventral striatum in anticipation of a reward (Tripp & Wickens, 2008).

Based on this information, recommendations for clinical practice are that children with an ADHD diagnosis may benefit most from immediate reward and need more frequent and consistent reinforcement than typically developing children for reward to be effective (Tripp & Wickens, 2008; Volkow et al., 2009, 2011). These recommendations have been integrated into behavioral interventions for children with ADHD (Van der Oord et al., 2008). As such, behavioral interventions, including the one in this study, typically contain similar evidence-based elements. Contingency management training, offered to parents and teachers, has been shown to be particularly effective. Child-sessions additionally incorporate reward contingencies and are used as motivation and stimulation for the training.

Despite the integration of these clinical recommendations, the utility of research findings has been limited in clinical practice, likely due to the highly heterogeneous nature of ADHD. Although at the group level ADHD has been linked to changes in reward sensitivity, these findings may not be relevant to all children with ADHD, due to large inter-individual differences (*Diagnostic and Statistical Manual of Mental Disorders*, 5th ed.; DSM-5; American Psychiatric Association [APA], 2013). This idea is underscored by the neurobiological heterogeneity found in the (dys)function of the ventral frontostriatal reward system (de Zeeuw et al., 2012; Durston et al., 2011; Lecei et al., 2019; Makris et al., 2009; Nigg & Casey, 2005). In all, due to the variability in reward processing, it may only be a relevant area of dysfunction for some children with ADHD. The modest effect of behavioral interventions on core symptoms of ADHD may be due to differences in sensitivity to reward in children with ADHD. For those children with greater sensitivity these interventions may be effective, while they are less so for children with less sensitivity. Therefore, we conducted a pilot study that aimed to use current knowledge about *individual* reward processing to predict the effectiveness of behavioral interventions for *individual* children. In addition, to test the construct validity of our reward sensitivity measures, we assessed correlations between the various measurement modalities.

In this study we attempted to use neuroscientific knowledge to inform clinical practice. We assessed whether individual measures of reward sensitivity could be used to predict the effectiveness of a behavioral intervention in reducing core-symptoms of ADHD (*Scoren met ADHD*; Schuurman et al., 2011). The intervention contained all evidence-based elements common to behavioral interventions in ADHD. We hypothesized that children with the greatest reward sensitivity would benefit most from the behavioral intervention.

## Method

### Study Design

The Medical Ethical Committee of the UMC Utrecht approved the study. Participant recruitment took place through the outpatient clinic of the Developmental Disorders Unit of the Department of Psychiatry (UMC Utrecht) and Altrecht Center for Mental Healthcare (Altrecht Jeugd, outpatient clinic for child and adolescent psychiatry in Utrecht). Data were collected at the UMC Utrecht.

Psychiatrists and clinical psychologists asked parents referred to the “Scoren met ADHD” (*Scoring with ADHD*) treatment program (Schuurman et al., 2011) for permission to share contact details with the study investigators. If granted, we contacted parents, provided them with information about the study and asked them to participate. We asked parents for written informed consent and invited participants to come to the UMC Utrecht with their parents to complete the pretreatment measurements. After completion of the behavioral intervention, we sent participants posttreatment questionnaires, which we asked them to complete at home.

### In/Exclusion Criteria

All participants had a clinical diagnosis of ADHD according to the *Diagnostic and Statistical Manual of Mental Disorders* (4th ed.; DSM-IV; APA, 1994) that was confirmed using the Diagnostic Interview Schedule for Children (DISC-IV, parent version; Shaffer et al., 2000). Participants were aged 8–13 years. Exclusion criteria for participation were: an estimated IQ below 80 (as the behavioral intervention is indicated only for children with an IQ above 80), any known cardiovascular or neurological disorder and insufficient parental command of either written or spoken Dutch, as we asked parents to fill in a number of questionnaires and complete a structured diagnostic interview. We did not consider psychiatric comorbidity or medication-use exclusion criteria, as we aimed for a typical clinical sample in a naturalistic setting.

### Participants

A total of 21 children with an average age of 9.9 years (range: 8.4–12.9) met full inclusion criteria for this study; 18 boys and three girls. We used DISC-IV scores to further classify participants. Children met criteria for the following DSM-IV subtypes: 13 combined type, one inattentive type, and seven hyperactive/impulsive type. We classified 12 participants as having comorbid oppositional defiant disorder (ODD), two of whom also met criteria for conduct disorder (CD). Nine participants were not receiving any psychopharmacological treatment, five were taking methylphenidate, one participant was receiving atomoxetine and the remaining six participants were taking other medication. Parents of one participant did not complete the posttreatment questionnaires. Consequently, the main outcome measures could not be computed for this participant and we excluded these data from all analyses.

### Intervention

Children participated in the behavioral intervention entitled “Scoren met ADHD” (*Scoring with ADHD*). This is a group-based behavioral therapy program for children with ADHD and their parents, that incorporates all major components of typical behavioral child/parent interventions in ADHD (Van der Oord et al., 2008). The name is based on the football-theme applied throughout the program. Group-sessions are fully protocolized and include approximately 4–6 children per group. Child sessions take place on a weekly basis over the course of 12 weeks, with concurrent biweekly parent sessions. The program also offers a teacher session, where teachers are informed about the program and instructed to apply similar techniques and language in the classroom as used during the intervention. Child sessions focus on developing impulse control and social problem-solving skills. A reward-contingency program is used as an incentive to work on assignments. Parent sessions focus on teaching parents to offer structure and effectively use reinforcement contingencies and on training them to help their child achieve better social problem-solving skills.

### Pre/Posttreatment Change

We measured pre/posttreatment change in ADHD symptoms using the Strengths and Weaknesses of ADHD and Normal Behavior (SWAN) rating scale (Lakes et al., 2012) administered before and after treatment. Parents rated the behavior of their child on 18 items based on the symptoms listed in the *DSM*-IV definition of ADHD. The SWAN is designed for parents to rate behavior relative to peers on a 7-point scale ranging from “far below average” via “below average,” “somewhat below average,” “average,” “somewhat above average,” “above average,” to “far above average.” We coded the scale from −3 to +3 and computed average scores for the inattention and hyperactivity subscales. Subsequently, we computed a change score by subtracting the pretreatment score from the posttreatment score. We computed a total change score by averaging the attentional and hyperactivity change scores.

### Reward Sensitivity Measures

We operationalized reward sensitivity using three modalities. Behaviorally, using a questionnaire, neuropsychologically, using two neuropsychological tasks and psychophysiologically, using heart rate measures. We used these measures to capture a variety of indices of reward-sensitivity and to assess preliminarily which of these modalities was most predictive of pre/posttreatment change.

#### Questionnaire

We used the Dutch translation of the Sensitivity to Punishment and Sensitivity to Reward Questionnaire for children (SPSRQ-C; Colder & O’Connor, 2004) as a parental rating of reward-related behavior. The Dutch version was validated by Luman et al. (2012). It has been shown to differentiate between typically developing children and children with ADHD, specifically on measures of sensitivity to reward. It contains 33 items scored on a 5-point rating scale. The Dutch translation of the questionnaire is best categorized by five factors: social fear, reward responsivity, impulsivity/fun seeking, drive, and punishment sensitivity. We averaged the items in each factor to compute overall factor scores. We expected the factors reward responsivity, impulsivity/fun seeking, and drive to be most closely related to the construct of reward processing and therefore used these as the main measures of reward sensitivity. The reward responsivity factor captures how excited and motivated a child is by reward. The drive factor has a strong social component and captures how motivated and competitive a child is to stand out or be the best. The impulsivity/fun seeking factor provides an index for risky, unfair or unwanted behavior to gain reward or social status. The name of the factor “impulsivity/fun seeking” suggests a relationship with the broader category of impulsive behaviors, and may lie on the continuum of that spectrum. However, this factor mostly captures impulsive negative behaviors intended to gain reward and may therefore be more closely related to reward sensitivity than impulsivity as intended in the core symptoms of ADHD. The two additional factors; social fear items and punishment sensitivity index anxiety and fearful behaviors. We included these two factors in post hoc analyses to assess their association with pre/posttreatment differences.

#### Neuropsychological tasks

We used two neuropsychological tasks to probe different aspects of reward processing. The Hungry Donkey Task (HDT; Crone & van der Molen, 2004) is a computerized child-friendly version of the Iowa Gambling Task (Bechara et al., 1994). This task is a hallmark in the field of decision-making and learning based on reward feedback and measures this specific aspect of reward sensitivity. The objective is to earn the Donkey as many apples as possible by repeatedly choosing one of four doors. There were two disadvantageous doors that resulted in high gains, but infrequently led to very high losses. The two other doors resulted in lower gains, but also in far less loss and were therefore advantageous overall. The task consisted of a total of 200 trials. Before administration of the HDT, we instructed children that they would receive their end score (number of apples won) in treats at the end of the testing session. The reward sensitivity measure derived from this task was the percentage of advantageous doors chosen throughout the task.

The Spongers task is a child-friendly version of the Monetary Incentive Delay Task (MID), where reward frequency and magnitude are experimenter-controlled (de Zeeuw et al., 2012; Knutson et al., 2001). This task probes for reward sensitivity by contrasting response times of rewarded trials to response times of unrewarded trials and has been shown to activate the ventral frontostriatal reward system (Durston et al., 2011). During the task, children saw a cue of a wallet containing either 0, 5, or 15 cents (reward magnitude), indicating the amount of money they could earn in the upcoming trial. Subsequently, the task required subjects to guess as fast as they could which of the two cartoon figures (SpongeBob or Patrick Star) was hiding the wallet containing the reward. If they guessed correctly, participants saw a thumbs up and the reward was added to their overall reward. If they guessed incorrectly, they saw a thumbs down and no money was added to the overall reward. To reinforce quick responses, the maximum response window was 1,250 ms. Participants received no reward for responses after this window. The task was rigged to produce two reward frequency conditions; two blocks where participants’ guesses were correct 80% of the time and two blocks where they were correct 20% of the time. Across the task, all participants earned a total of €10, which they received in the form of a gift-certificate at the end of the task. Response times are a measure of approach behavior, as earlier studies using this task have shown that faster response times occur for higher rewards, an effect that is attenuated in ADHD (van Hulst et al., 2015). We used response time differences between reward magnitude conditions (0 cents vs. 5 or 15 cents) as reward sensitivity measures. This was computed using a regression procedure designed to limit the effect of response time variability (as de Zeeuw et al., 2012). The manipulation of both reward magnitude (2×) and reward frequency (2×) resulted in four outcome measures: RegB_20_1 (difference between response times in 0 and 5 cent trials in blocks with a 20% reward frequency), RegB_20_2 (difference between response times in 0 and 15 cent trials in blocks with a 20% reward frequency), RegB_80_1 (difference between response times in 0 and 5 cent trials in blocks with an 80% reward frequency), and RegB_80_2 (difference between response times in 0 and 15 cent trials in blocks with an 80% reward frequency). These regression coefficients indicate the level of reward sensitivity; if the regression coefficient is smaller than 1, the response times in the reward condition are faster than the response times in the nonreward conditions, hence reward has a stronger influence on performance, indicating reward sensitivity.

#### Physiological measures

The measurement of physiological responses to reinforcement have a long history in research of disruptive disorders (Fowles, 1980; Luman et al., 2005; Matthys et al., 2013; Raine, 1996). They have been used to study the fearlessness-hypothesis of more anti-social disorders (oppositional defiant disorder or conduct disorder). Differences in heart rate variability in response to reward between normally developing participants and participants with ADHD have frequently been described. (Bubier & Drabick, 2008; Crone et al., 2003; Crowell et al., 2006; Iaboni et al., 1997; Luman et al., 2007). As such, we decided to include heart rate measures as a commonly used psychophysiological proxy of reward sensitivity. We measured heart rate (with a two-lead electrocardiogram) using the Vrije Universiteit—Ambulatory Monitoring System (VU-AMS) (de Geus et al., 1995) as a marker for physiological response to reward. We recorded heart rate data during a baseline period and during both tasks as the inter-beat-interval (IBI) in milliseconds. We found a number of gaps in the data sets of three participants due to temporary signal losses in heart rate recordings. Therefore, we individually checked all data sets and structured them to ensure that all available data could be used. Subsequently, we replaced outlying IBIs from the data sets, defined as IBIs shorter than 400 ms, longer than 1,500 ms or over three standard deviations away from the average IBI of that individual’s own data set. We set the baseline period as 90 IBIs prior to the instruction-phase of the first task. We computed heart rate variability (HRV) as the root mean square of successive differences (RMSSD) in IBIs during baseline, the HDT, total Spongers task, Spongers 20% frequency blocks and Spongers 80% frequency blocks. Similarly, we computed average heart rate (AHR) in each of these conditions. For both AHR and HRV, we computed difference scores by subtracting baseline values from task values and by subtracting values in the Spongers 20% reward condition from values in the Spongers 80% reward condition. These variables will be referred to as HRV_HungryDonkey-Baseline_, HRV_Spongers-Baseline_, HRV_Spongers80%−Spongers20%_, AHR_HungryDonkey-Baseline_, AHR_Spongers-Baseline_, AHR_Spongers80%−Spongers20%_.

### Statistical Analysis

#### Quality checks and pre/posttreatment change

We tested all variables for missing values, normality of distribution (using the Shapiro-Wilk test) and outliers (using an interquartile range larger than three). Two participants missed a question on the pretreatment SWAN questionnaire and three participants missed a question on the posttreatment SWAN questionnaire. We omitted these scores when computing average scale scores. The majority of the data was normally distributed and included no univariate outliers. For some measures, we ran regression analyses with non-normally distributed data, and additionally tested these using spearman correlations. This was the case for the SPSRQ-C scales Reward Sensitivity and Drive, the percentage advantageous doors in the HDT, and the HRV data. Since the nonparametric analyses for these measures did not differ meaningfully from the parametric ones, we report only the parametric analyses. To assess pre/posttreatment change we used paired samples *t* tests to compare parent-rated symptoms of ADHD before and after treatment.

#### A-priori reward sensitivity and pre/posttreatment change

We tested for associations between reward sensitivity measured prior to treatment and pre/posttreatment change in ADHD symptoms. To do so, we used linear regression with SWAN change scores as outcome variables (pre/posttreatment change in attention, hyperactivity, and total scores) and reward sensitivity measures as predictor variables. Primary predictor variables included: the SPSRQ-C subscales Reward Sensitivity, Impulsivity/Fun Seeking and Drive; the HDT percentage advantageous doors, Spongers RegB_20_2, Spongers RegB_80_2, HRV_Hungry Donkey-Baseline_, HRV_Spongers-Baseline_, and HRV_Spongers80%−Spongers20%_. In exploratory, post hoc analyses we entered a number of additional reward sensitivity variables. We entered heart rate measures (AHR_Hungry Donkey-Baseline_, AHR_Spongers-Baseline_, and AHR_Spongers80%−Spongers20%_) and two additional factors of the SPSRQ-C questionnaire (social fear and punishment sensitivity) as predictor variables.

#### Multi-method correlational analyses of reward sensitivity measures

To assist hypothesis-generation for future research, we tested associations between the reward sensitivity measures in different modalities, using correlational analyses. Further information on these analyses can be found in the supplementary material.

#### Sensitivity Analyses

We ran sensitivity checks for the influence of IQ and baseline heart rate on our main analyses. As some of the original heart rate data sets included temporary signal losses, we assessed whether the quality of heart rate data influenced the results. We did this by entering a dummy variable of heart rate data quality (complete data sets vs. incomplete data sets) in the regression analyses with significant results. We found no evidence of these measures affecting our outcome and therefore reported our results without them.

## Results

### Pre/Posttreatment Change

Values of pre- and posttreatment measurements of the SWAN questionnaire can be found in Table 1. Parent-rated symptoms of ADHD (measured by the SWAN-questionnaire) differed significantly between pre- and posttreatment. Parents rated their children as less inattentive (*M* = −.60, *SD* = 1.01) and less hyperactive (*M* = −.72, *SD* = .79) after treatment, than they did before treatment, *M* = −1.29, *SD* = .78 and *M* = −1.47, *SD* = .67 respectively; *t*(19) = −3.59, *p* < .05 and *t*(19) = −3.29, *p* < .05.

**Table 1.** Demographic Variables and SWAN Scores.

Demographic variables			
*N*	Boys/Girls	Age	Age range
21	18/3	9.9	8.4–12.9
Parent-rated symptoms of ADHD			
SWAN-score	Overall	Hyperactivity	Attention
Pretreatment	−1.38	−1.47	−1.29
Posttreatment	−0.66	−0.72	−0.60
Change-score	0.72	0.76	0.69

*Note.* SWAN = Strengths and Weaknesses of ADHD and Normal Behavior; ADHD = attention-deficit/hyperactivity disorder; *N* = number of participants.

### A-Priori Reward Sensitivity and Pre/Posttreatment Change

**Figure 1.**
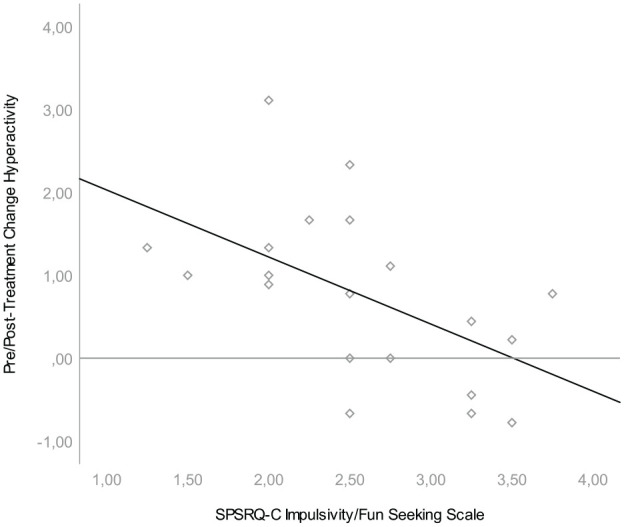
Association between SPSRQ-C Impulsivity/Fun Seeking Scale and pre/posttreatment difference in SWAN hyperactivity. *Note.* Association between SPSRQ-C scale and pre/posttreatment difference in SWAN hyperactivity. Children who scored lower on the Impulsivity/Fun Seeking Scale, showed a larger pre/posttreatment difference. SPSRQ-C = Sensitivity to Punishment and Sensitivity to Reward Questionnaire for Children; SWAN = Strengths and Weaknesses of ADHD and Normal Behavior.

**Figure 2.**
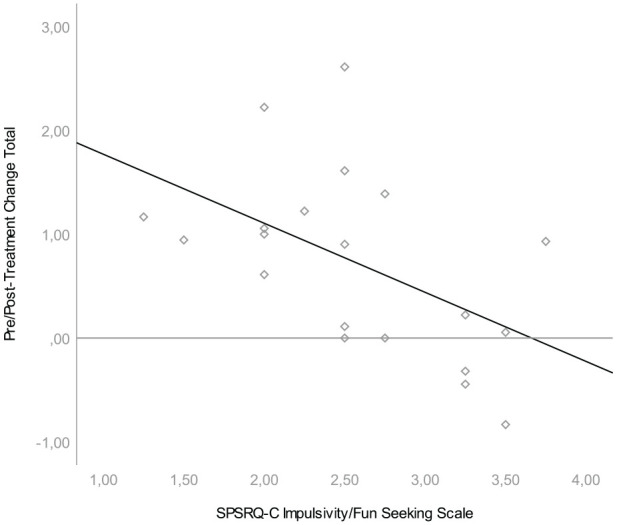
Association between SPSRQ-C Impulsivity/Fun Seeking Scale and pre/posttreatment difference in SWAN total. *Note.* Association between SPSRQ-C scale and total SWAN pre/posttreatment difference. Children who scored lower on the Impulsivity/Fun Seeking Scale, show a larger pre/posttreatment difference. SPSRQ-C = Sensitivity to Punishment and Sensitivity to Reward Questionnaire for Children; SWAN = Strengths and Weaknesses of ADHD and Normal Behavior.

We found that pre/posttreatment change was associated with one scale of the parent-rated reward sensitivity questionnaire. Lower scores on the SPSRQ-C Impulsivity/Fun seeking scale were associated with larger change scores on both the SWAN Hyperactivity Scale, *β* = −.809, *t*(20) = −2.717, *p* = .014, and the SWAN Total Scale, *β* = −.665, *t*(20) = −2,551, *p* = .020; see Figures 1 and 2. Effect sizes for both associations were large (*r* = .54 and *r* = .52, respectively). This means that hyperactive behavior was more likely to diminish during treatment in children with low parental ratings on the impulsivity/fun seeking scale. The impulsivity scale consists of four slightly divergent items that focus on gaining social status through unfair means, not being able to resist the temptation to do forbidden things, showing risky behavior to get a reward and not doing things you enjoy so as to not be rejected or criticized. We found no relationship between pre/posttreatment change in ADHD symptoms and task performance, heart rate variability or the other SPSRQ-C subscales. In an exploratory, post hoc regression analyses we found no relationship between pre/posttreatment change in ADHD symptoms and heart rate measures (i.e., AHR_Hungry Donkey-Baseline_, AHR_Spongers-Baseline_, AHR_Spongers80%−Spongers20%_) or the two additional SPSRQ-C sub-scales. Results of these regression analyses can be found in Table 2.

**Table 2.** Regression Analyses of Reward Sensitivity Measures and Pre/Posttreatment Differences.

	SWAN attention			SWAN hyperactivity			SWAN total		
	*F*	Sign.	ES (*r*)	*F*	Sign.	ES (*r*)	*F*	Sign.	ES (*r*)
Primary analyses									
SPSRQ-C Reward Responsivity	0.104	.751	.076	0.306	.587	.129	0.026	.873	.038
SPSRQ-C Impulsivity/Fun Seeking	3.725	.070	.414	7.384*	**.014**	**.539**	6.508*	**.020**	**.515**
SPSRQ-C Drive	0.191	.667	.102	0.442	.514	.155	0.029	.867	.040
HDT percentage advantageous doors	0.002	.968	.009	0.541	.471	.171	0.196	.663	.104
RegB_20_2	0.000	.985	.005	0.030	.863	.041	0.008	.928	.022
RegB_80_2	0.003	.954	.014	1.137	.300	.244	0.333	.571	.135
HRV_Spongers-Baseline_	0.002	.964	.011	0.880	.361	.216	0.313	.583	.131
HRV_Hungry Donkey-Baseline_	0.566	.461	.175	0.047	.831	.051	0.056	.816	.056
HRV_Spongers80%−Spongers20%_	0.952	.342	.224	0.480	.497	.161	0.773	.391	.203
Average heart rate analyses									
AHR_Spongers-Baseline_	0.910	.353	.219	2.435	.136	.345	1.880	.187	.308
AHR_Hungry Donkey-Baseline_	0.293	.595	.127	1.575	.226	.284	0.974	.337	.227
AHR_Spongers80%−Spongers20%_	4.422	.050	.444	2.133	.161	.325	3.546	.076	.406

*Note.* SWAN = Strengths and Weaknesses of ADHD and Normal Behavior; ADHD = attention-deficit/hyperactivity disorder; ES = effect size; *r* = Pearson’s correlation; SPSRQ-C = Sensitivity to Punishment and Sensitivity to Reward Questionnaire for children; HDT = Hungry Donkey Task; RegB_20_2 = difference between response times in 0 and 15 cent trials in blocks with a 20% reward frequency; RegB_80_2 = difference between response times in 0 and 15 cent trials in blocks with an 80% reward frequency; HRV = heart rate variability; AHR = average heart rate.

### Correlations Between Different Measures of Reward Sensitivity

In correlations between variables of the same modalities, we found different heart rate measures to be highly positively intercorrelated, with only a few significant correlations in the questionnaire and task data. Across modalities, measures of reward sensitivity mostly did not correlate. Further information on these correlations is provided in the supplementary materials.

## Discussion

The aim of this pilot study was to assess whether reward sensitivity, as assessed a-priori, can predict pre/posttreatment differences in core ADHD symptoms in the context of a behavioral intervention using reward contingencies. Our treatment was a reward contingency assisted behavioral intervention incorporating evidence-based elements of typical behavioral interventions. We hypothesized that those children with ADHD who had the greatest sensitivity to reward, would benefit most from the behavioral intervention. To test this hypothesis, we collected reward sensitivity data from different modalities; a questionnaire, two neuropsychological tasks and heart rate measurements, and assessed their predictive relationship with pre/posttreatment differences in parental ratings of ADHD symptoms. Children showed improvement overall, as parents rated their children less inattentive and hyperactive after treatment. These findings are in line with previous research that has shown that behavioral interventions are moderately effective (Daley et al., 2014; Sonuga-Barke et al., 2013). Children who had low parental ratings of reward sensitivity, specifically on the impulsivity/fun seeking scale of the SPSRQ-C, improved most during treatment. No other measure of reward sensitivity predicted pre/posttreatment change in ADHD symptoms.

The name of the scale “impulsivity/fun seeking”, was introduced during previous iterations of factor analyses of the SPSRQ-C (Colder & O’Connor, 2004; Luman et al., 2012), but perhaps does not fully represent the four items in the Dutch version of this scale (Luman et al., 2012). These items are related to accomplishing social status through unfair means, not being able to resist the temptation to do things that are forbidden, showing risky behavior to get a reward and avoiding rejection and criticism by not doing things that are considered fun. The scale measures impulsive negative behavior to gain (social) reward, even if through unfair means. It may be argued that there is overlap between this subscale and the broader spectrum of impulsive behaviors, on which the impulsive behaviors described in the criteria for ADHD also fall. However, we found only low to moderate and not-significant correlations between this subscale and ADHD symptoms as measured by the SWAN-questionnaire pretreatment. Moreover, the impulsivity/fun-seeking subscale appears predictive of pre/posttreatment change in our data, even when forcing pretreatment SWAN measures into the analysis as a covariate. The impulsivity/fun seeking subscale can be understood in terms of the reward and punishment sensitivity theory by Gray and McNaughton (2000) which describes a behavioral activation system (BAS) and a behavioral inhibition system (BIS). Focusing on reward while ignoring the negative associations with or consequences of actions may be in line with a predominantly active BAS in combination with an inactive BIS. This imbalance has previously been described in children with a diagnosis of ODD (Matthys et al., 2004; Newman & Wallace, 1993).

Although the association between impulsive negative behaviors to gain reward and the effectiveness of treatment is promising, these results should be interpreted with caution. First, in view of the exploratory nature of our study, we chose not to correct for multiple testing. This increases the likelihood of false positive findings. Moreover, both measures were based on parental ratings. The lens through which parents view and rate their children may affect outcome as questionnaire data completed by the same rater are not entirely independent. This phenomenon has been called common method variance (Richardson et al., 2009). Its impact on outcome is inherently difficult to assess, but a suggested solution to detecting common method variance is employing numerous different measurement modalities when gathering data. In this study, we did in fact include a number of reward sensitivity measures that did not rely solely on the parental perspective. However, we found no associations between these measures and pre/posttreatment change. In sum, both common method variance and increased likelihood of false positive findings may play a part in our results. As such, these findings should be taken as an incentive for further research with larger samples, rather than as definitive knowledge on (children with) ADHD.

In addition to the matters discussed above, there is the initial value-problem: those children who show the most severe symptoms on pretreatment measures may be most likely to improve, whereas children with milder symptoms may show less improvement. We carried out additional analyses where we defined treatment outcome simply as posttreatment SWAN scores. These analyses showed similar results to the ones in the main paper, although two additional regression analyses reached statistical significance: the SPSRQ-C Impulsivity/Fun Seeking scale was associated not only with the posttreatment SWAN Hyperactivity Scale, but also with the posttreatment SWAN Attention Scale. Furthermore, we found that differences in heart rate variability between the Spongers 80% condition and the Spongers 20% condition were associated with the posttreatment SWAN Hyperactivity scale. Detailed information on these analyses can be found in the supplementary material.

All our measures of reward sensitivity have previously been used successfully in research on reward sensitivity. The SPSRQ-C-questionnaire is the most ecologically valid measure, commonly used in research on reward sensitivity. It has been shown to differentiate between typically developing children and children with an ADHD diagnosis (Luman et al., 2012). Our two neuropsychological tasks have been extensively used in the literature on reward sensitivity. The Spongers task is a relatively direct measure of children’s response time to reward, whereas the HDT is a proxy of decision-making and learning based on reward feedback. Similarly, our psychophysiological measures (heart rate and heart rate variability) have been shown to relate to reward processing and to differentiate between typical and clinical populations (Bubier & Drabick, 2008; Crone et al., 2003; Crowell et al., 2006; Iaboni et al., 1997; Luman et al., 2007). Although all of these measures have been related to reward processing in various ways, it is not clear whether they measure one and the same construct. We found only few correlations between the different operationalizations of reward sensitivity (parent-rated reward sensitivity, task-based reward sensitivity, and heart-rate data). This is in line with other studies noting that different measures of theoretical constructs often have no or very low correlations (Fuermaier et al., 2015; Potvin et al., 2016; Toplak et al., 2013). One potential explanation for this lack of associations can be found in concerns about the assumption that such different modalities measure the same underlying concept. This assumption is usually made after various (objective and subjective) measures of a construct show differences between clinical and typical populations, differences that are assumed to prove construct validity (Barkley & Fischer, 2011; Biederman et al., 2008; Tucha et al., 2011, 2009). For example, this is the case for executive functioning, where task-based measures and parental rating scale measures are implicitly thought to measure the same underlying construct, but in fact have few meaningful correlations (Toplak et al., 2013). The same may be the case for the measures of reward sensitivity in the current study. Faridi et al. (2015) remarked that construct labels attached to instruments or scales may lead to the unjust assumption that there is overlap in constructs measured by different instruments. In all, it is uncertain if the different measures of reward sensitivity used, tapped into one overall construct of reward processing.

We found no evidence for the predictive value of heart rate measures or neuropsychological task performance on pre/posttreatment change. Other studies have previously tried to identify and quantify clinically relevant biomarkers of reward sensitivity (Fosco et al., 2018; Luman et al., 2007; Tripp & Wickens, 2008; van de Wiel et al., 2004; Volkow et al., 2001). To date, there is no evidence for robust links between neurobiological markers and clinically relevant outcome measures. Finding common ground between the clinical and neuroscientific fields as such, is proving to be difficult, as needs for specificity and reliability in measures differ across the fields. Moreover, both the tendency of clinical psychology to adjust slowly to paradigm shifts and the lack in neurosciences of employing clinically relevant methods (Beauchaine et al., 2008) result in limited interchangeability between the fields (Pine, 2011). A mere call from the field of neuroscience to apply its insights in clinical practice ignores the need to think about which neuroscientific contribution would be most meaningful and helpful in clinical practice. The relevance of heavily controlled neuroscientific research in a clinical environment, that is inherently strongly influenced by context, is not a given. Reward sensitivity might be a clinically relevant measure if we can better delineate when, where and how it can be meaningfully measured in clinical practice and whether it can inform treatment choices independent of individual patient context.

### Limitations

This study was setup as a pilot study and accordingly, we included only a small number of participants. As such, our power to detect more subtle differences in reward sensitivity was limited. Furthermore, we used a single-arm design and lacked a control condition for pre/posttreatment change. Therefore, it is difficult to discern if pre/posttreatment differences are due to the behavioral intervention or whether other factors played a role. Due to the small-scale design of our study, we did not assess the impact of psychopharmacological treatment on the relationship between reward sensitivity and treatment outcome. This may be an important factor to study more thoroughly in future studies.

### Conclusion

In this pilot study, we made a first attempt to connect the extensive neuroscientific literature on reward processing in children with ADHD to clinical practice. We found that pre/posttreatment change was associated with one specific aspect of parent-rated pretreatment reward sensitivity. This result suggests that children with low impulsive negative behavior to gain reward, may benefit most from a behavioral intervention using reward contingencies. This preliminary finding is promising in that it suggests individual neuropsychological profiles in ADHD may perhaps be applicable for predicting the effectiveness of treatments. However, this is a small pilot study and larger studies are warranted before translating these findings to everyday clinical practice.

## Supplemental Material

Supplement_material – Supplemental material for Which Child Will Benefit From a Behavioral Intervention for ADHD? A Pilot Study to Predict Intervention Efficacy From Individual Reward Sensitivity Click here for additional data file. Supplemental material, Supplement_material for Which Child Will Benefit From a Behavioral Intervention for ADHD? A Pilot Study to Predict Intervention Efficacy From Individual Reward Sensitivity by Myrte J. M. van Langen, Branko M. van Hulst, Miriam Douma, Maarten Steffers, Nicolle M. H. van de Wiel, Els van den Ban, Sarah Durston and Patrick de Zeeuw in Journal of Attention Disorders
