# Supplementary material for: Which Child Will Benefit From a Behavioral Intervention for ADHD? A Pilot Study to Predict Intervention Efficacy From Individual Reward Sensitivity
Source: J Atten Disord. 2020 Jun 11;25(12):1754–64. doi: 10.1177/1087054720928136 (PMC8404726; doi:10.1177/1087054720928136)
Supplement: Supplement_material – Supplemental material for Which Child Will Benefit From a Behavioral Intervention for ADHD? A Pilot Study to Predict Intervention Efficacy From Individual Reward Sensitivity [file Supplement_material.pdf]

Supplementary Material

Additional Outcome Measure- Post-Treatment SWAN

The definition of treatment outcome as pre-post treatment change has a potential pitfall, often coined the initial value problem. Children who are more 'impaired' have more room to improve, whereas children who are closer to the norm, are less likely to reach similar levels of improvement, because of a lack of room for improvement. In order to account for this problem, we carried out additional analyses where we defined treatment outcome as the post-treatment score. Although this definition also has flaws (individual differences in pre-treatment scores, carrying over into post-treatment scores may now unjustly be correlated with reward sensitivity), we wanted to provide an overview of the results for comparison.

The additional analyses, using SWAN Attention and SWAN Hyperactivity post-treatment scores, can be found in supplemental table 1. The majority of the analyses did not reach statistical significance, similar to the analyses with pre-post treatment change scores. We found three associations between reward-sensitivity measures and SWAN post-treatment scores. The SPSRQ-C Impulsivity/Fun Seeking scale is now associated not only with the post-treatment SWAN Hyperactivity Scale, but also the post-treatment SWAN Attention Scale. Lower scores on the SPSRQ-C Impulsivity/Fun seeking scale were associated with higher scores on both the post-treatment SWAN Hyperactivity Scale (β = -.589, $t(20)$ = -2.522, p= .021) and the post-treatment SWAN Attention Scale (β = -.959, $t(20)$ = -3,626, p= .002). In addition, we found that a larger difference in heart rate variability between the Spongers 80% condition and the Spongers 20% condition, was associated with higher scores on the post-treatment SWAN Hyperactivity scale (β = .054, $t(20)$ = 2,750, p= .013).

Supplemental Table 1. *Regression Analyses of Reward Sensitivity Measures and Post Treatment ADHD symptoms*

	SWAN Attention Post-treatment			SWAN Hyperactivity Post-treatment		
	F	Sign.	ES (r)	F	Sign.	ES (r)
Primary Analyses						
SPSRQ-C Reward Responsivity	.390	.540	.146	.103	.752	.075
SPSRQ-C Impulsivity/Fun Seeking	<u>13.149</u>	<u>.002*</u>	<u>.650</u>	<u>6.361</u>	<u>.021*</u>	<u>.511</u>
SPSRQ-C Drive	1.838	.192	.304	.702	.413	.194
HDT Percentage Advant. Doors	.242	.629	.115	2.793	.112	.366
RegB_20_2	.266	.612	.121	.105	.750	.076
RegB_80_2	.272	.608	.122	.406	.532	.148
$HRV_{Spongers-Baseline}$	.485	.495	.162	.366	.553	.141
$HRV_{HungryDonkey-Baseline}$	.107	.748	.077	.466	.503	.159
$HRV_{Spongers80\%-Spongers20\%}$	.695	.415	.193	<u>7.563</u>	<u>.013*</u>	<u>.544</u>
Average Heart Rate Analyses						
$AHR_{Spongers-Baseline}$	.251	.622	.117	.453	.510	.157
$AHR_{HungryDonkey-Baseline}$	.024	.878	.037	.010	.921	.024
$AHR_{Spongers80\%-Spongers20\%}$	3.231	.089	.390	3.251	.088	.391

ADHD, Attention-Deficit/Hyperactivity Disorder; SWAN, Strengths and Weaknesses of ADHD and Normal Behavior; ES, Effect Size; r, Pearson's Correlation; SPSRQ-C, the Sensitivity to Punishment and Sensitivity to Reward Questionnaire for children; HDT, Hungry Donkey Task; RegB_20_2, difference between response times in 0 and 15 cent trials in blocks with a 20% reward frequency; RegB_80_2, difference between response times in 0 and 15 cent trials in blocks with an 80% reward frequency, HRV, Heart Rate Variability; AHR, Average Heart Rate.

Correlations between different measures of reward sensitivity

We tested for correlations between the various measures of reward sensitivity (SPSRQ-C factor scores, spongers performance measures, HDT performance measures, heart rate data and heart rate variability data). We used Spearman correlations for analyses of non-normally distributed variables (the SPSRQ-C scales Reward Sensitivity and Drive, the percentage advantageous doors in the HDT, and the HRV data) and Pearson correlations for all normally distributed data. Statistically significant correlations were further explored in scatterplots to identify bivariate outliers. If present, we re-ran the analyses without the outliers to assess their effect on the model.

Results of all correlational analyses can be found in Supplemental Table 2. Correlations between reward sensitivity measures were split into two categories: correlations between variables from the same modality (within questionnaires, behavioral task data, or heart rate data) and correlations between different modalities. Within the same modalities, we found heart rate data to be highly positively correlated, and only few significant correlations in the questionnaire and task data. Across modalities, measures of reward sensitivity mostly did not correlate. Out of the 67 associations we tested, four were found to be nominally significant**.** Questionnaire data did not correlate with any of the other measures, nor did the behavioral measures of the HDT and Spongers task. We found only few correlations between behavioral measures and physiological data (e.g. HDT and task data).

Supplemental Table 2. *Correlation Table for Reward Sensitivity Measures.*

			1.	2.	3.	4.	5.	6.	7.	8.	9.	10.	11.	12.	13.	14.
1	SPSRQ-C Reward Responsivity	Cor	-	,014	**,740****	-,317	-,183	,029	,215	,202	,005	-,027	,032	,038	,047	-,085
		Sig.	-	,954	**,000**	,173	,441	,904	,362	,394	,982	,909	,894	,874	,844	,722
2	SPSRQ-C Impulsivity/Fun Seeking	Cor		-	-,087	-,190	-,148	-,134	-,078	,276	,437	,166	-,269	,115	-,084	-,183
		Sig.		-	,716	,422	,534	,575	,744	,239	,054	,484	,251	,630	,725	,441
3	SPSRQ-C Drive	Cor			-	-,170	-,214	,149	,189	-,116	,121	,015	,190	,173	,060	,233
		Sig.			-	,472	,364	,531	,425	,625	,613	,949	,421	,466	,802	,322
4	SPT RegB_20_1	Cor				-	**,529***	,129	,131	,169	-,287	-,286	,038	-,027	,043	,029
		Sig.				-	**,016**	,588	,581	,477	,220	,222	,875	,911	,858	,904
5	SPT RegB_20_2	Cor					-	-,127	,191	,225	-,101	-,084	,277	-,108	,034	,194
		Sig.						,594	,419	,340	,673	,724	,238	,652	,886	,412
6	SPT RegB_80_1	Cor						-	**,492***	-,029	,195	,289	-,065	,394	**,508***	-,321
		Sig.						-	**,028**	,905	,409	,217	,787	,086	**,022**	,168
7	SPT RebB_80_2	Cor							-	-,152	,236	,194	**,538***	,331	**,561***	,158
		Sig.							-	,522	,316	,413	**,014**	,155	**,010**	,506
8	HDT	Cor								-	-,174	-,131	**-,587****	-,263	-,123	-,135
		Sig.								-	,463	,582	**,006**	,262	,604	,571
9	$HRV_{Spongers-Baseline}$	Cor									-	**,770****	,185	**,669****	**,565****	,173
		Sig.									-	**,000**	,435	**,001**	**,009**	,466
10	$HRV_{HungryDonkey-Baseline}$	Corr										-	,259	**,674****	**,836****	,068
		Sig.										-	,271	**,001**	**,000**	,777
11	$HRV_{Spongers80\%-Spongers20\%}$	Cor											-	,105	,398	,362
		Sig.											-	,659	,082	,116
12	$AHR_{Spongers-Baseline}$	Cor												-	**,637****	,132
		Sig.												-	**,003**	,578
13	$AHR_{HungryDonkey-Baseline}$	Cor													-	-,099
		Sig.													-	,679
14	$AHR_{Spongers80\%-Spongers20\%}$	Cor														-
		Sig.														-

This table shows all correlations between reward sensitivity measures. Spearman correlations are in the gray boxes and Pearson correlations are in the white boxes. Significant correlations within the same measurement-modalities are in bold. Significant correlations between different measurement modalities are in bold and underlined. SPSRQ-C, the Sensitivity to Punishment and Sensitivity to Reward Questionnaire for children; SPT, Spongers Task; RegB_20_1, difference between response times in 0 and 5 cent trials in blocks with a 20% reward frequency; RegB_20_2, difference between response times in 0 and 15 cent trials in blocks with a 20% reward frequency; RegB_80_1, difference between response times in 0 and 5 cent trials in blocks with an 80% reward frequency; RegB_80_2, difference between response times in 0 and 15 cent trials in blocks with an 80% reward frequency; HDT, Hungry Donkey Task; HRV, Heart Rate Variability; AHR, Average Heart Rate.
